# The Comparison of the Body Composition of Children at the Early School Age from Urban and Rural Area in Southwestern Poland

**DOI:** 10.1155/2018/9694615

**Published:** 2018-09-04

**Authors:** Agnieszka Chwałczyńska, Tomasz Rutkowski, Grzegorz Jędrzejewski, Dorota Wójtowicz, Krzysztof A. Sobiech

**Affiliations:** ^1^Department of Human Biology, University School of Physical Education, Al. Paderewskiego 35, 51-612 Wroclaw, Poland; ^2^Jan Długosz University, Częstochowa, Poland; ^3^Department of Physiotherapy, Public Higher Medical Professional School in Opole, Opole, Poland; ^4^Department of Physiotherapy, University School of Physical Education, Al. Paderewskiego 35, 51-612 Wroclaw, Poland

## Abstract

The aim of the study was to compare the segmental body composition with the use of fat–fat-free (FFF) index in children at early school age, depending on sex and place of residence, with particular emphasis on urban and rural areas. The study consisted of 329 children aged 7.78 (SD = 0.88; mean age in years). The study group was divided according to the place of residence and sex. The height and body mass, Body Mass Index (BMI), segmental body composition (Tanita BC-418MA), and FFF were calculated. A more frequent occurrence of excessive body weight was observed in children from rural areas (over 20%) compared to their peers from the urban area (10%). Statistically significant lower values of FFF index as well as in the lower limbs and torso were observed in the case of the examined children from the urban area as compared with their peers from the rural areas. The body composition of children living in metropolitan and rural areas is diverse. Lower values of FFF indexes were found in children from the city than in children living in villages. There are more children in the city with signs of being underweight and of normative body mass and paradoxically more overweight ones in the village.

## 1. Introduction

The problem of children's excess weight and obesity in Poland is becoming increasingly more serious. Already over 20% of children aged 7-10 years can be classified as overweight or obese [1–10] (Wolnicka et al., 2011). According to the latest global surveys, in 2014 the problem of excess weight and obesity has affected over 41 million children over the age of 5. According to studies carried out in Poland as part of the fourth edition of the “European Childhood Obesity Surveillance Initiative”, as many as 30% of 8-year-olds have an over-normative body weight based on World Health Organization (WHO) recommendations (BMI for age) [11, 12]. This problem not only applies to highly developed countries, but also includes younger and younger children. According to UNICEF data, the percentage of overweight and obese children under the age of 5 increased during the first decade of the 21st century from 11% to 19% [13, 14]. According to studies carried out by Freedman et al., as part of the Bogalusa Heart Study (2007), 53-90% of children having overweight under 6 years of age will have overweight and obesity in adulthood [14, 15].

Rolland-Casher et al. [16] described the phenomenon of so-called “adiposity rebound” characterized by an increase in body weight at about the age of 8 and this persistence continues until the end of the growing process [14, 16, 17].

Determination of body weight abnormalities or diagnosing excess weight or obesity in a child is based on a measure of body fat based on height and weight that applies to Body Mass Index (BMI). Although relatively precise in the case of people under the age of 18, thanks in part to the developed centile grids, the method omits the issue of abnormalities in the body's composition [18, 19]. Over the years, attempts have been undertaken to clarify the BMI by combining it with a standardized 50 percentile (Cola index) or average BMI for a given population [7, 20]. An attempt to evaluate body weight may be the BAI proposed by Bergman et al. [21], taking into account hip circumferences, but it is mainly directed towards adults and does not take into account the age differences or adipose tissue level. However, none of these methods included body composition [7, 21].

One of the most frequently used methods of assessing the components of the body is the electrical bioimpedance method that uses the resistance of tissues in the body under study: the BIA method. Based on simple devices, it allows you to estimate the percentage of adipose tissue, fat-free, and water in the body [22–24]. More accurate methods, dual energy X-ray absorptiometry (DXA), computed tomography, and magnetic resonance, allow the precise assessment of the body composition not only generally, but also segmentally [25]. The possibility of using these methods is limited mainly due to diagnostic needs in the case of medical orders; however, they are often expensive and rarely available [7] (Rutkowski et al., 2017).

Thanks to the multiple electrodes used in the BIA study, the human body is divided into 5 segments, denoting the composition of the body independently for each of them. The use of this method allows estimating changes that occur under the influence of directional physical activity. The assessment of segmental body composition is based on fat-skin fold measurements or on the photo-optic method [24]. However, these methods either are burdened with a researcher's error or determine the local composition of the body. Not only does wider use of segmental body composition assess changes, but in order to diagnose excess weight as obesity, the determination of limit values for body fat is required. Their application requires population studies for a given age and sex [7, 19].

Methods presented so far are based on height and body weight and, on the other side, on the percentage of body fat which does not fully reflect the problem of body mass abnormalities. The proportion of the areas of the human body seems more important. For a more complete diagnosis, indicators based on components of body mass were developed. Szczawinska et al. [26] in her MT (muscular-fat) index relied on the mass of lean tissue in relation to adipose tissue [26]. This method developed limits values for normative body weight, overweight, and obesity: I°, II°, and III°. However, the age of the person examined is not taken into account and neither is the general fat tissue value as it is calculated from an algorithm based on measurements of skinfolds, which requires precision and experience in the application of this methodology [26]. The modernized form of this index was presented by Mialich et al. based on fat tissue determined by the bioimpedance method and BMI as the BMIfat index [27, 28]. The reuse of the weight-increase ratio does not give a full picture of the distribution of body fat, while not differentiating the age of the examined person. The index based on the body composition is the fat–fat-free (FFF) index, which is the quotient of the basic components of the human body, showing the relationship between these components. Adapted to age, it can be a tool for assessing body mass depending on the age and sex of the subjects. Due to the use of segmental body composition, the fat–fat-free segment index can also be determined [7].

## 2. The Purpose of the Study

The purpose of the study was to compare the segmental body composition with the use of fat–fat-free (FFF) index in children in the early school age (7-10 years old), by sex and place of residence, with particular emphasis on the urban and rural areas.

## 3. Research Material and Methods

The research project was conducted with the permission of the Commission of Bioethics at the Wroclaw Medical University No. 487/2006 of November 2, 2006. The research was carried out and financed as part of statutory research conducted as part of employment at the School Physical Education in Wroclaw.

The study assessed 329 children aged between 7 and 10 years from an urban (Wroclaw, n = 169) and rural area (Lewin Brzeski n = 160). The mean age of the children was for Wroclaw 7.69 ± 0.79 years and for Lewin Brzeski 7.88 ± 0.97 years. The height and weight were determined, the BMI was calculated, and the BMI age for a given age and sex was determined in the examined group using the OLAF calculator [2.4]. The examined children did not differ in terms of age and body height. There were statistically significant differences in terms of body weight, BMI, and BMI percentile. Children from the urban area were lighter and had a lower BMI and a lower BMI percentile. The study group was divided according to the place of residence and sex as follows: GU, girls from Wroclaw, urban area (n = 86); GR, girls from Lewin Brzeski, rural area (n = 89); BU, boys from Wroclaw, urban area (n = 80); BR, boys from Lewin Brzeski, rural area (n = 71).

In the examined groups, the occurrence of nonfatal body mass was determined on the basis of the BMI for age percentile, determined using a calculator developed as part of the OLAF project ([1], http://olaf.czd.pl/index.php?option=com_content&view=article&id=103:calculator). The WHO classification was applied, taking into account the age and sex of the respondent, distinguishing

- underweight: BMI <5 percentile,
- normative body weight: 5 percentile <BMI <85 percentile,
- overweight: BMI> 85 percentile.

In the children that were examined, segmental body composition was determined by means of using the Tanita (Japan) eight-electrode body composition analyzer BC-418MA. The total and segmental values of adipose tissue in percent (FatP, [%]), fat mass (FatM, [kg]), and fat-free (FFM, [kg]) were estimated including right lower limbs (leg) (RL), left limbs (leg) (LL), upper right limbs (arm) (RA), left limbs (arm) (LA), and trunk (TR). With the use of fat and nonfat component, the FFF (fat–fat-free) index was calculated using the formula

(1) $$\begin{array}{c} FFF\left(\;N\;\right)=\frac{FatM\left(N\right)\left[kg\right]}{FFM\left(N\right)\left[kg\right]} \end{array}$$

  N is the index order according to the body segment,
  FatM (N) is the segmental fat mass [kg],
  FFM (N) is the segmental fat-free mass [kg],
  FFF is the fat–fat-free index: FFF1 fat–fat-free index for the right leg, FFF2 fat–fat-free index for the left leg, FFF3 fat–fat-free index for the right arm, FFF4 fat–fat-free index for the left arm, and FFF5 fat–fat-free index for the trunk [7, 8] (Rutkowski et al., 2017).

In order to compare the out-of-school activities of the examined children, a questionnaire was carried out regarding regular, organized physical activity outside the school, Physical Activity Questionnaire-Children (PAQ-C) [29]. The results of the PAQ-C were slightly lower in the group of children from rural area (mean: 2.8 points on a 5-point scale) in relation to the respondents from urban area (mean: 3.1 points). The PAQ-C was supplemented with questions about the most frequently chosen forms of physical activity. Surveys regarding personal data and physical activity (PAQ-C, complemented by sports disciplines) were completed by the parents of the examined children as a qualifying element for the research project. In the urban area about 80% of the children would typically undertake additional physical activities, more often in the form of swimming classes (50% of 8-year-old children and 70% of 9-year-olds), karate (45% of respondents), or football (37% boys). In the rural area, only 10% of the children participated in nonschool forms of physical activities by choosing between karate and a football section addressed to children.

In order to obtain the results, a statistical analysis was carried out using the Statistica 13 program (StatSoft program). Statistical analysis was based on descriptive statistics of the average and those presenting the standard deviation from the sample. To make a comparison of the examined children,* t*-test (Student's* t*-distribution) tested for independent groups was used. The nonparametric test for independent groups, the Kolmogorov-Smirnov test, was used for intergroup comparisons. The significance level was set at 0.05.

## 4. Results

In the studied group, the girls from the rural area are statistically significantly heavier, have a higher BMI and BMI for age percentile, and have a larger fat and nonfat component in comparison to girls from the urban area. The examined boys were not statistically significantly different in terms of their height, BMI percentile, and nonfat tissue mass; the remaining values were statistically significantly higher in the group of boys from the rural area. The results are shown in Table 1.

**Figure 1:**
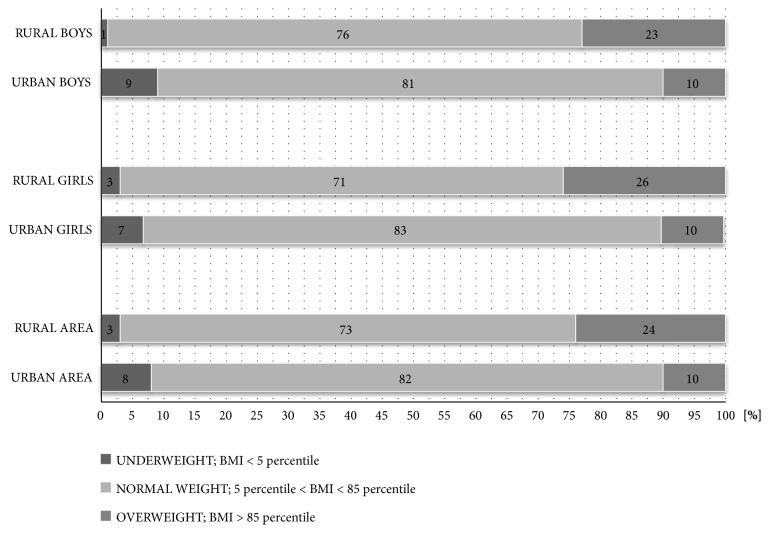
Occurrence of body mass abnormalities in the examined children depending on the place of residence.

On the basis of BMI percentiles, more than twice the prevalence of excess weight in the examined children from the rural area was found compared with children from the urban area. The number of children on the BMI classification is shown in Figure 1.

Comparing the segmental body composition, a statistically significant higher fat and fat-free components were found in all body segments except for the left upper limb in girls from the rural area compared to girls from the urban area. The percentage of segmental adipose tissue does not differ significantly in the examined children, irrespective of their gender and the place of residence. In the boys' group, the mass of the segmented fat component is statistically significantly different and is higher in the examined children from the rural area. The results of the segmental body composition are presented in Table 2.

**Figure 2:**
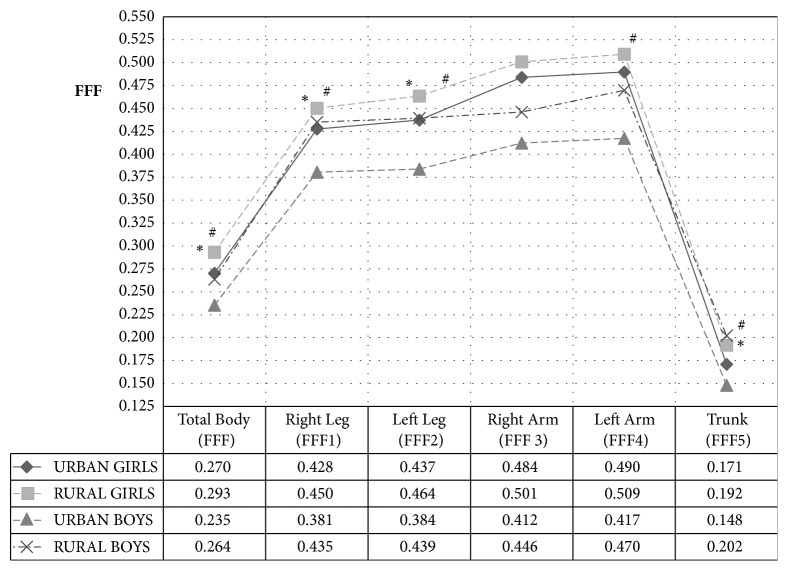
Fat–fat-free (FFF) index in the examined children depending on the place of residence. (*∗* indicates differences statistically significant between girls from urban area and girls from rural area; # indicates differences statistically significant between boys from urban area and boys from rural area).

The value of the FFF index is the highest in the case of the examined girls from rural areas and the lowest in the boys from urban areas (Figure 2). The highest fat-free indexes were observed in the upper limbs, but, only in the case of boys from rural areas, differed significantly between left and right limb (p <0.01). Comparing segment and general indicators, statistically significant differences were found for each segment, irrespective of the their sex and place of residence of the respondent. Similar differences exist between the trunk indexes and peripheral indicators. No differences were observed apart from the group of girls from the rural area in the right and left limb and the right upper limb and boys from the same area.

Comparing the FFF index depending on the place of residence, statistically significant differences were found in girls in the general indicator, in both lower limbs and torso, whereas in the boys differences were found in all indicators except for the right upper limb (Figure 2).

In order to determine the distribution of body fat, the percentage of adipose tissue in individual segments was calculated in relation to the total percentage of adipose tissue according to the following formula.

(2) $$\begin{array}{c} \%Segmental\;\;FatP=\frac{\left(segmental\;\;FatP*100\%\right)}{global\;\;FatP} \end{array}$$

**Figure 3:**
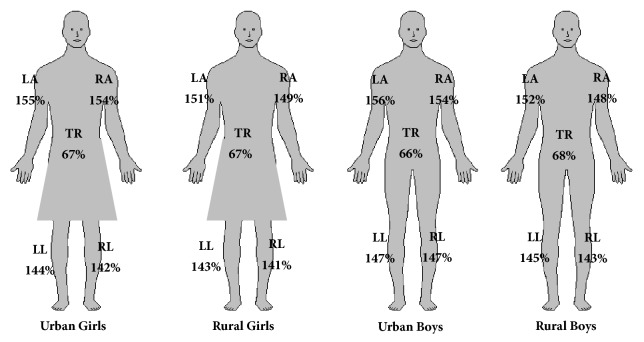
Percentage distribution of adipose tissue in examined children depending on sex and place of residence.

On the basis of the conducted tests, distal distribution of adipose tissue was found in the examined children (Figure 3). Statistically significant differences were recorded in the case of upper limbs between those examined from urban and rural area for both sexes. There were no statistically significant differences in the distribution of adipose tissue depending on the sex of the subjects, which may indicate a lack of sexual dimorphism in terms of body composition, including age.

## 5. Discussion

The composition of the human body changes with ontogenetic development. These changes vary depending on the age and sex of the subjects. In the progressive period of ontogenesis (before sexual maturity), a dynamic increase of fat mass as well as fat-free mass resulting from body growth can be observed. In the adult period, the height of the body is stabilized, and the mass and thus the composition of the body depend on the lifestyle and diet of the person. In the infertile period (after 40/50 years of age), the body height decreases physiologically while the percentage of adipose tissue increases [7]. The intensity of the changes is determined by genetic factors and influenced as well by environmental factors. In the process of ontogenesis, moments determining the possibility of body mass abnormalities can be observed [7, 19]. At the age of about 2 years, there should be a physiological reduction in the percentage of adipose tissue associated with the increase in motor activity of the child. The opposite process, called by some people the critical moment of development of the adipose tissue or “rebound obesity” associated with the onset of puberty, is the age of 7-10 years. During this period, the child's physical activity depends primarily on the parents, while the habits that affect the subsequent quality of life are shaped [7, 14, 15, 19].

A child 7-10 years is overseen by compulsory education, based on a similar educational program. However, the “value” of physical education classes at school depends largely on the size of the area in which the institution is located. The presented research project compared the urban centers where access to various forms of physical activities is full but only limited by the financial possibilities of the parents and the interests of the child as well as the rural amenities proposing to its inhabitants a poor offer of activities in the spectrum of physical activity. The previous belief that children from rural centers are more physically active compared to their peers from the city was based on outdated assumptions. Currently, rural centers provide, regardless of parents, direct access to school from their place of residence, which is not provided in urban areas. At the same time, technological development—Rural Development Program for 2014-2020 (RDP 2014-2020)—enabled the use of unlimited access to the Internet, which resulted in children spending a longer time in front of their computer monitor or TV.

The difference between the rural and urban areas has been preserved in terms of access to organized forms of physical activities, especially in the field of extracurricular activities. In the research conducted, the possibilities of using classes addressed to children in the context of out-of-school clubs were compared. In the examined urban areas, the vast majority of children (80%) undertook additional physical activity according to their interests such as football, swimming, dancing, and horse riding. However, in the rural area, only 10% of the children declared willing participation in additional physical activities, in two available sports, football and karate.

Insufficient physical activity can consequently lead to body mass abnormalities [19]. The confirmation of this statement may be the number of children from the rural area, whose abnormal body weight was found on the basis of the tests and the BMI for age percentile mark. As part of the nationwide OLAF project carried out in 2007-2010, the excess weight was found to be in 15-20% of children in early school age [1, 4]. Similar results, depending partly on the research area, were obtained by Felińczuk and Hama, Mleczko and Szmigiel, and Chwałczyńska et al. [2, 3, 8]. By analyzing in detail the studied group, a large variation in the incidence of excess weight was observed, depending on the place of residence of the children. In the studied group from the urban area, only 10% showed a BMI above 85 percentile, without regard to gender, while there were almost one and a half more children from the rural area in this group (♀ 26%, ♂ 22.5%). Similar results of the incidence of excess weight and obesity in the Opolskie Voivodeship (Lewin Brzeski) were obtained by researchers comparing the results of the “Fruit at School” program. They drew attention to a large percentage (27%) of third-grade students in the Opolskie Voivodeship, who were overweight and obese, while the problem of obesity was more significant in the case of boys rather than girls [30].

The presented abundances of groups with body mass abnormalities were based on the weight-increase index, BMI, but do not take into account the internal structure of the human body (components of the body composition, fat and lean mass with muscle mass), which has a decisive influence on the physical fitness of the examined children.

By examining children at early school age from various areas, urban and rural ones, there were statistically significant differences in terms of body weight and the amount of adipose tissue. The examined children from the rural area have a higher body mass, higher fat mass (FatM), and a nonfat mass (FFM) compared to their peers from metropolitan area. When comparing children, even in early school age, sexual diversity, which at this stage of development is just beginning to reveal itself, should be taken into account. After applying the sex division, it is worth noting that the boys from the rural area differ statistically significantly from the boys from the city in terms of the fat components, while their mean percentage of general body fat indicates the presence of hidden excess weight.

The problem of excess weight in the group of younger school children can be determined not only by body weight. Increasingly, the assessment of general and segmental body composition can be included in the standards for the assessment of the occurrence of excess body weight and obesity. In the studies on the body composition of girls and women, the differences were statistically significant in terms of fat distribution depending on the ages that were found [7]. The presented project obtained similar results confirming peripheral distribution of adipose tissue in girls and boys. Comparing the distribution of the fat component based on the segmental analysis of the body composition, it was found that the highest amount is found in the upper limbs. Considering the overall percentage of adipose tissue as 100%, it is possible to determine the obesity of the trunk in the examined children within 67%, while in the lower limbs at about 143%. (Figure 3).

The presented percentage distribution of adipose tissue (Figure 3) shows slight differences between girls and boys. It is connected with the ontogenetic period in which the respondents—all the examined subjects—were in the prepubertal period [31]. This may indicate a lack of sexual differentiation in the distribution of adipose tissue in the younger school age. At the same time, a disproportion of fat distribution was observed in all the groups and segments on the left side of the body FatP are larger in comparison to the right side. It may be related to the right-sidedness of the examined children and, consequently, greater muscle mass on the dominant side. Presentation of the proportion of fat distribution can be used, inter alia, for postural control. The person examined on the body composition analyzer takes an unstrained body position and does not correct their posture by muscle tension. Another application may be to control the course of the training process, but in this case the evaluation is only partial; it does not include the more important component, that is, the weight of lean tissue for sportsmen.

In the presented studies, there were no statistically significant differences in the percentage of adipose tissue (FatP) between children from urban and rural areas; these differences were noticed when fat mass was compared (FatM). The higher fat mass was found in girls and boys from a rural center and this resulted in their higher overall body weight compared to children from the city. This testifies to a smaller mass of nonfat (FFM) (muscle) children from rural areas, which in turn leads to lower physical fitness. Less lean mass may result from less frequent physical activity declared by parents of children from rural areas. The higher adipose tissue weight may indicate the presence of latent overweight or predisposition to the appearance of overweight and obesity in adulthood. The existence of 14% and 16% (Figure 2) more children from rural areas with overweight confirms the problem of overweight in rural areas.

The latest body composition research shows that not only does the level of percentage of body fat determine the occurrence of body mass abnormalities, but also the ratio of fat to lean mass is more important [7, 26–28]. In the research conducted, a fat–fat-free index was used to assess the distribution of body weight in girls and boys. Comparing the FFF index in girls, depending on the abnormalities of body mass and place of residence, higher index values were found in those examined from the rural area compared with the inhabitants of the inner city. As in the Chwałczyńska study [7], the highest values were recorded in the upper limbs [7, 8]. The FFF index in the examined girls from the rural area was on mean higher by 0.03 compared to the group from the urban area.

The values of the FFF index in the boys' group are lower than those in the group of girls of the same age. This indicates a lower body fat mass in relation to the nonfat mass than in the case of girls. Boys from a rural area have a higher FFF index compared to their peers from the inner city. In the conducted research, it was observed that the boys from the village area determined by means of the FFF indicator resemble more girls than boys from an urban area. This may indicate later sexual differentiation of rural children compared to their peers from the city. Differences in fat–fat-free structure in the boys are most visible in the lower limbs and the right upper limb; the FFF index is statistically significantly lower in boys from the urban area. A large percentage (23%) of children with excess body weight in a rural area show the need to increase the amount of physical education classes for this age group. The problem of excess weight and obesity concerns not only the early school period, but also adolescence. In research which was carried out on middle school students from the rural area, there was a significant reduction in physical fitness, with a disproportionate increase in body mass represented by BMI and an increase in the percentage of total body fat in some of the examined pupils; even body weight of above 40% was observed as normal for the age ♀ up to 29% and ♂ up to 21% [32, 33].

Owing to the limited space and personnel possibilities in smaller towns, the school should be a sports and educational center where children and parents can enhance their knowledge about a healthy lifestyle and participate in more sports activities. As the results from the research are presented, the amount of children's physical activity, especially in rural areas, is insufficient, which produces a negative effect in the form of a positive energy balance and, consequently, excess weight or obesity. Emergency programs at schools run by therapists, aimed at weight reduction as well as correction of coexisting abnormalities, for example, body posture, physical fitness, or eye-hand coordination, could be a way out of the situation [20, 23].

In the present study, fat–fat-free (FFF) index was used for the first time in relation to such a large group of boys. So far, the results of women and girls have been presented [7, 34], in addition to changes in the indicator under the influence of targeted physical activity in children with overweight and obesity [8]. The FFF index showed the significance of the general and segmental assessment of body composition in the case of developmental age in the classification of body mass abnormalities.

## 6. Conclusions

The following conclusions can be drawn on the basis of the results obtained:

1. The body composition of children living in the urban and rural areas is diverse. The subjects were found to have significant disproportions in the percentage of adipose tissue depending on the segment or general intake.
2. Lower general and segmental fat–fat-free (FFF) index values were found in children from urban area than in the ones living in rural area.
3. There are more children in the city with underweight and normative body mass and more overweight ones in the village. The same regularity is observed when the respondents are divided according to gender. There will be much more overweight girls and boys in the rural area than in the urban one.
4. Thanks to the use of fat-free index, statistically significant higher fatness can be observed in the relations between children living in the rural area and from urban area. The applied FFF index turned out to be more sensitive than BMI to determine differences between particular groups.

## Figures and Tables

**Table 1:** Anthropometric data of the studied children from the urban and rural areas.

	Urban Girls (GU) n = 89 Mean ± SD	Rural Girls (GR) n = 89 Mean ± SD	p	Urban Boys (BU) n = 80 Mean ± SD	Rural Boys (BR) n = 71 Mean ± SD	p
Height [cm]	126.77 ± 7.35	128.62 ± 7.80	p = 0.11	129.01 ± 6.73	130.35 ± 7.67	p = 0.25

Weight [kg]	25.69 ± 5.38	27.77 ± 5.77	p = 0.01*∗*	27.27 ± 5.37	29.55 ± 7.78	p = 0.04#

BMI [kg/m^2^]	15.85 ± 2.15	16.66 ± 2.36	p = 0.02*∗*	16.24 ± 1.99	17.18 ± 3.10	p = 0.03#

BMI for age percentile	45.39 ± 29.94	54.65 ± 30.17	p = 0.04*∗*	46.99 ± 27.43	54.25 ± 28.27	p = 0.11

Adipose tissue in percent (FatP) [%]	21.10 ± 3.67	22.44 ± 4.03	p = 0.02*∗*	18.89 ± 3.64	20.50 ± 5.10	p = 0.02#

Fat Mass (FatM) [kg]	5.57 ± 2.14	6.41 ± 2.40	p = 0.01*∗*	5.30 ± 1.97	6.41 ± 3.65	p = 0.02#

Fat – free mass FFM [kg]	20.13 ± 3.46	21.36 ± 3.62	p = 0.02*∗*	21.98 ± 3.62	23.15 ± 4.39	p = 0.07

*∗* Differences statistically significant between girls from urban and rural area. # Differences statistically significant between boys from urban and rural area.

**Table 2:** The segmental body composition of the examined children from urban and rural areas.

	Urban Girls (GU) n = 89 Mean ± SD	Rural Girls (GR) n = 89 Mean ± SD	p	Urban Boys (BU) n = 80 Mean ± SD	Rural Boys (BR) n = 71 Mean ± SD	p
Right leg (RL)						
FatP [%]	29.82 ± 3.45	30.96 ± 4.14	p = 0.09	27.34 ± 3.38	28.58 ± 5.00	p = 0.07
FatM [kg]	1.30 ± 0.45	1.47 ± 0.48	p = 0.02*∗*	1.27 ± 0.46	1.63 ± 1.11	p = 0.01#
FFM [kg]	3.01 ± 0.63	3.23 ± 0.68	p = 0.02*∗*	3.28 ± 0.79	3.53 ± 1.00	p = 0.08

Left leg (LL)						
FatP [%]	30.18 ± 3.41	31.47 ± 4.21	p = 0.06	27.47 ± 3.42	28.81 ± 4.97	p = 0.06
FatM [kg]	1.29 ± 0.44	1.46 ± 0.48	p = 0.01*∗*	1.25 ± 0.46	1.59 ± 1.08	p = 0.01#
FFM [kg]	2.91 ± 0.59	3.11 ± 0.64	p = 0.03*∗*	3.18 ± 0.77	3.42 ± 0.97	p = 0.10

Right arm (RA)						
FatP [%]	32.23 ± 3.42	32.76 ± 4.38	p = 0.44	28.75 ± 3.98	29.26 ± 4.72	p = 0.48
FatM [kg]	0.37 ± 0.13	0.42 ± 0.15	p = 0.03*∗*	0.34 ± 0.12	0.41 ± 0.25	p = 0.02#
FFM [kg]	0.76 ± 0.18	0.82 ± 0.18	p = 0.01*∗*	0.82 ± 0.19	0.89 ± 0.25	p = 0.05

Left arm (LA)						
FatP [%]	32.50 ± 4.15	33.32 ± 5.17	p = 0.31	29.27 ± 3.88	30.26 ± 4.93	p = 0.17
FatM [kg]	0.39 ± 0.16	0.43 ± 0.16	p = 0.09	0.37 ± 0.13	0.46 ± 0.28	p = 0.01#
FFM [kg]	0.79 ± 0.18	0.84 ± 0.18	p = 0.05	0.88 ± 0.21	0.94 ± 0.25	p = 0.08

Trunk (TR)						
FatP [%]	14.39 ± 3.90	15.63 ± 4.87	p = 0.11	12.70 ± 3.78	13.93 ± 4.69	p = 0.08
FatM [kg]	2.23 ± 1.01	2.64 ± 1.15	p = 0.01*∗*	2.09 ± 0.91	3.05 ± 3.14	p = 0.01#
FFM [kg]	12.70 ± 1.93	13.38 ± 1.99	p = 0.02*∗*	13.85 ± 1.71	14.34 ± 1.96	p = 0.11

*∗* Differences statistically significant between girls from urban and rural area. # Differences statistically significant between boys from urban and rural area.

## Data Availability

The results of the research will be available on request from interested persons (query: agnieszka.chwalczynska@awf.wroc.pl) or from the corresponding author. This limitation is related to the principles of personal data protection and the conditions we have received as part of the approval of the Bioethical Commission. The data will be free of sensitive data, including the date of birth (age will be available at the time of the examination), name, and surname (the coded number of the respondent will be available).
